# Ligature-induced periodontitis exacerbates high-carbohydrate/high-fat diet-induced fatty liver in mice under non-diabetic conditions

**DOI:** 10.1186/s12903-026-07992-6

**Published:** 2026-03-04

**Authors:** Misako Tari, Mikihito Kajiya, Tsuyoshi Fujita, Kazuhisa Ouhara, Tomoyuki Iwata, Shinji Matsuda, Hidemi Kurihara, Noriyoshi Mizuno

**Affiliations:** 1 Department of Periodontal Medicine, Hiroshima University, 1-2-3 Kasumi, Minami-Ku, Hiroshima, 734-8553 Japan; 2 Innovation & Precision Dentistry, Hiroshima University, 1-2-3 Kasumi, Minami-Ku, Hiroshima, 734-8553 Japan

**Keywords:** Fatty liver, High-carbohydrate diet, Non-alcoholic fatty liver disease, Periodontitis

## Abstract

**Background:**

Recent studies have suggested that periodontitis affects non-alcoholic fatty liver disease (NAFLD). Until now, the underlying mechanisms have mainly been investigated in the context of diabetes, which is a risk factor for NAFLD. However, clinical research indicates that 80% of NAFLD patients do not have diabetes, with a complication rate of 40% even in advanced cases. In this study, we investigated whether periodontitis exacerbates NAFLD under non-diabetic conditions using a high-carbohydrate/high-fat diet (HCHFD).

**Methods:**

We first developed non-diabetic NAFLD mice induced by an HCHFD and then analyzed the effect of ligature-induced periodontitis in an established NAFLD mouse model. Mice were fed a normal chow (NC), an HCHFD, or a high-fat diet (HFD), a commonly used diet in previous NAFLD studies for 6 weeks.

**Results:**

HCHFD induced hepatic steatosis without causing glucose intolerance or insulin resistance, whereas a HFD showed glucose intolerance accompanied by hepatic steatosis. Ligature-induced periodontitis facilitated HCD-induced steatosis and caused interlobular fibrosis in the liver.

**Conclusion:**

A non-diabetic mouse model for NAFLD, closer to the clinical presentation than pre-existing mouse models, indicated that ligature-induced periodontitis exacerbated non-diabetic NAFLD, which accounts for the majority of the NAFLD population.

**Supplementary Information:**

The online version contains supplementary material available at 10.1186/s12903-026-07992-6.

## Background

In recent decades, non-alcoholic fatty liver disease (NAFLD) has become the most common chronic liver disease worldwide, with an estimated prevalence as high as 32% in the global population [1]. It has a wide range of pathologies, from non-alcoholic fatty liver (NAFL) to non-alcoholic steatohepatitis (NASH) [2]. NASH is a more serious condition of NAFLD with fibrosis which progresses from 10–20% of NAFL cases. Furthermore, 3% of NASH cases also progress to cirrhosis and hepatocellular carcinoma [3]. Among the risk factors related to the development of NAFLD, diabetes is regarded as the most dominant one [4]. It is also important to note that the term NAFLD, though it is still widely used, has been criticized over its vague definition. Some researchers say that its nomenclature is misleading, giving the impression that it does not coexist with other liver diseases, which deviates from the actual pathology [5]. The alternative term, metabolic-associated fatty liver disease (MAFLD), was suggested in 2020 by an international panel of experts and steadily taking its place [6]. This alternative term implies that the metabolic dysfunction is the main driver of this liver disease [7].

Periodontal disease is a chronic inflammatory disorder caused by the host immune response to oral pathogenic bacteria, which results in the destruction of the alveolar bone and connective tissues around the teeth [8]. The prevalence of periodontitis in dentate adults is estimated to be approximately 62% [9]. Previous studies have shown that periodontitis can be a source of pathogenic microorganisms and inflammatory cytokines throughout the body, affecting distant organs. Periodontitis is widely accepted to be linked to several systemic diseases, including diabetes, preterm birth, and rheumatoid arthritis [10–12].

Yoneda et al. were the first to demonstrate that periodontitis could be a risk factor for the development of NAFLD [13]. Their study was followed by several other studies investigating the mechanisms underlying NAFLD using mouse models. These studies reported mechanisms that involve, or at least coexist with, diabetes, employing a high-fat diet, which inevitably induces diabetes along with NAFLD in a short period of time [14–18]. It has long been indicated that periodontitis exacerbates diabetes and that diabetes and NAFLD are closely correlated [19–21]. Thus, many studies have focused on glucose intolerance or insulin resistance induced by periodontitis and the exacerbation of NAFLD [15, 17], which seems reasonable since diabetes is the most dominant exacerbating factor of NAFLD [4]. However, from an epidemic point of view, only 20% of all NAFLD cases are complicated by diabetes, with a complication rate of 40%, even in NASH [3]. Furthermore, the potential existence of an NAFLD exacerbating pathway that does not involve obesity or insulin resistance has been reported [22]. Thus, an NAFLD mouse model that corresponds to the clinical presentation would be required to assess the influence of periodontitis and other risk factors in the majority of patients with NAFLD. Establishing this model enables us to investigate whether periodontitis directly exacerbates NAFLD in the absence of diabetes.

A high-fat diet provides a large amount of saturated fatty acids and accumulates lipids in various tissues, resulting in the simultaneous onset of diabetes and fatty liver disease. On the other hand, excess carbohydrates ingested from food reach the liver at first and then activate the lipid synthesis pathway, thereby causing fatty liver [23]. In this context, diabetes occurs after the establishment of a fatty liver. Moreover, High-fat diets which have been used in previous studies regarding causal relationship between periodontitis and NAFLD contained larger percentages of fat (as measured in calories) and smaller percentages of carbohydrate compared to that of general NAFLD patients [24]. High-fat diets used in the previous studies are generally around 60% fat and 26% carbohydrate, while typical diet of NAFLD patients are around 36% fat and 46% carbohydrate [24]. Therefore, we hypothesized that a high-carbohydrate/high-fat diet, but not a high-fat diet, could reproduce the general diet among patients with NAFLD and thus induce fatty liver disease (i.e., NAFLD) in mice without diabetes.

This study aimed to establish an NAFLD mouse model with no diabetes symptoms using a high-carbohydrate/high-fat diet and to investigate whether experimental periodontitis exacerbates NAFLD, regardless of diabetes.

## Methods

### Feed

Commercially available HFD-32 was provided by Japan CLEA (Tokyo, Japan). HFD-32 contains 506.7 kcal/100 g (35.88 g fat and 27.43 g carbohydrates). The high carbohydrate/high-fat diet (HCHFD) was produced by Oriental Yeast Co., Ltd (Tokyo, Japan) with minor modification of “western-diet,” which is frequently employed for the study of atherosclerosis using apolipoprotein E-deficient mice [25]. This feed was developed to contain 450 kcal/100 g (21 g fat and 54.15 g carbohydrate). MF (359 kcal/100 g) was purchased from Oriental Yeast Co., Ltd. and used as a normal chow diet (NC). Details of the HFD and HCHFD are shown in Table 1. Percentages of fat and carbohydrate (as measured in calories) of each diet are shown in Table 2.

**Table 1:** Ingredients in HCHFD and HFD32

MF		HCHFD		HFD32	
Formula(g/Kg)					
Water	81	Sucrose	341.46	Crystalline Cellulose	55
Crude Protein	232	Corn Starch	115	Maltodextrin	82.5
Crude Fat	49	Cellulose	50	Lactose	69.28
Crude Ash	59	Unsalted butter	245	Sucrose	67.5
Crude Fiber	33	Cholesterol	1.5	Powdered beef tallow	158.8
Nitrogen Free Extracts	547	Casein	195	Safflower oil(high oleic acid)	200
		DL-Methionine	3	Milk casein	245
		Mineral Mix, AIN-96	35	Egg white	50
		Calcium Carbonate	4	L-cystein	4.3
		Vitamin Mix, AIN-93	10	AIN93 vitamin mix	14
		Tertiary butylhydroquinone	0.04	AIN93G mineral mix	50
				Choline bitartrate	3.6
				Tertiary butylhydroquinone	0.02
Energy(kcal/100 g)					
355.7		450		507.6

**Table 2:** Percentages of fat and carbohydrate (as measured in calories)

	**MF**	**HCHFD**	**HFD32**
Fat	13%	42%	60%
Carbohydrate	62%	42.7%	26.6%

### Animals

This study used 6-week-old male BALB/cAJcl mice (Charles River Laboratories Japan, Yokohama, Japan), which are reportedly less likely to develop glucose intolerance than C57BL/6 mice [26]. The experimental protocol was approved by the Animal Care Committee of Hiroshima University (protocol no. A19-139). Mice were fed a NC, HFD, or HCHFD for 6 weeks (*n* = 5/group). The body weight was monitored weekly. Food was weighed and exchanged daily. Animals were fasted 16 h for the intraperitoneal glucose tolerance test (IPGTT). For the insulin tolerance test, animals were fasted for 6 h. After fasting, the mice were intraperitoneally injected with glucose (2 g/kg) or insulin (1U/kg) (Humulin R; Eli Lilly and Company, Indianapolis, IN, USA). The blood glucose concentrations were determined using a glucometer (Syntron Bioresearch, Carlsbad, CA, USA). Subsequently, livers were obtained for histological analyses.

### Macroscopic and histological analyses of the liver

After capturing photographs of the isolated livers for macroscopic analysis, samples collected from the left hepatic lobes were fixed with 4% paraformaldehyde overnight. After washing, the specimens were dehydrated using a graded ethanol series, cleared with xylene, and embedded in paraffin. Lastly, 4-μm-thick sections were processed with hematoxylin and eosin (HE) or Azan-Mallory. Evaluation of staging of liver fibrosis was conducted by a blinded pathologist.

Samples for Oil red-O staining were collected from the left hepatic lobe, fixed with 4% paraformaldehyde overnight, and placed successively in 10%, 20%, 30%, and 40% sucrose overnight. The tissues were embedded in an Optimal Cutting Temperature compound (Sakura Finetek, Tokyo, Japan). Then, 7-μm-thick sections were cut using a cryostat and then stained with Oil red-O and counterstained with hematoxylin. The lipid area was quantified using ImageJ software.

### A ligature-induced experimental periodontitis mouse model

Ligature-induced periodontitis was induced according to a previously described method [27]. Briefly, a 5–0 silk ligature was placed around the second maxillary molar on both sides in mice until the end of the experiment under anesthesia with medetomidine (0.3 mg/kg), midazolam (4 mg/kg), and butorphanol tartrate (5 mg/kg). The animals were fed NC or HCHFD (*n* = 5/group). The body weight was monitored weekly. Six weeks after ligature placement, IPGTT or ITT was conducted, and the maxilla and liver were excised for analysis. The livers were examined as described above.

### Histological analysis of periodontal tissue

Periodontal tissues were fixed overnight in 4% paraformaldehyde. They were then decalcified with 10% ethylenediaminetetraacetic acid (EDTA) for one week, dehydrated using graded ethanol, cleared with xylene, and embedded in paraffin. Then, 5-µm-thick sections were cut in the coronal plane and processed by hematoxylin and eosin staining.

### Measurements of triglyceride and total cholesterol in liver

Liver tissues were homogenized in chloroform/methanol (2:1, v/v), and lipid extracts were prepared using the Folch method [28]. Intrahepatic cholesteroland triglyceride levels were measured using the enzymatic assay kits Cholestest CHO and Cholestest TG (Sekisui Medical, Tokyo, Japan) respectively.

### Measurements of serum lipid and liver damage markers

Venous blood samples were obtained from the orbital veins of mice following protocols approved by the Animal Care Committee of Hiroshima University, and serum was isolated by centrifugation. Levels of total cholesterol, free cholesterol, triglyceride, low-density lipoprotein cholesterol, high-density lipoprotein cholesterol, AST, and ALT in serum samples were assessed by Oriental Yeast Co., Ltd (Tokyo, Japan).

### RNA preparations and quantitative real-time PCR

Total RNA was extracted from liver tissue using the RNA iso plus (TaKaRa Bio, Shiga, Japan). The extraction method was performed according to the manufacturer’s instructions.One μg total RNA was reverse-transcribed to cDNA using ReverTra Ace (Toyobo, Osaka, Japan).Real-time PCR was performed using Light Cycler and THUNDERBIRD Next SYBR qPCR Mix (Toyobo, Osaka, Japan) to assess the relative mRNA expression levels of IL-1β, TNF-α, TGFβ, Acc1, Glck. the Thermal PCR conditions were determined according to the manufacturer’s protocol. Specifically, the PCR thermal profile consisted of initial denaturation at 95 °C for 10 min, followed by 40 cycles at 95 °C for 15 s and 60 °C for 1 min. Gene expression levels were normalized to those of the reference gene, *GAPDH*. PCR primers used in the study are listed in Table 3.

**Table 3:** Sequence of primers

Gapdh	Forward: CCTGGAGAAACCTGCCAAGTATG
	Reverse: TGTTGCTGTAGCCGTATTCATTGT
Acc1	Forward: ACACCATGTTGGGAGTTGTG
	Reverse: GCTGTTCCTCAGGCTCACAT
Glck	Forward: TATGAAGACCGCCAATGTGA
	Reverse: TTTCCGCCAATGATCTTTTC
TGFβ	Forward: GACCGCAACAACGCCATCTAT
	Reverse: CGAAAGCCCTGTATTCCGTCTC
IL-1β	Forward: CCTTGTGCAAGTGTCTGAAGC
	Reverse: TCATCTTTTGGGGTCCGTCAAC
TNF-α	Forward: CTATGTCTCAGCCTCTTCTC
	Reverse: CATTTGGGAACTTCTCATCC

### Statistical analysis

Experiments were repeated more than three times, and the values are expressed as the means ± standard deviation (SD). Statistical analysis was performed using a two-tailed unpaired Student’s *t*-test to compare two groups. One-way ANOVA was performed for multiple group comparisons using the Tukey–Kramer post-hoc test.

## Results

### HCHFD induced non-diabetic NAFLD in BALB/cAJcl mice

**Fig. 1:**
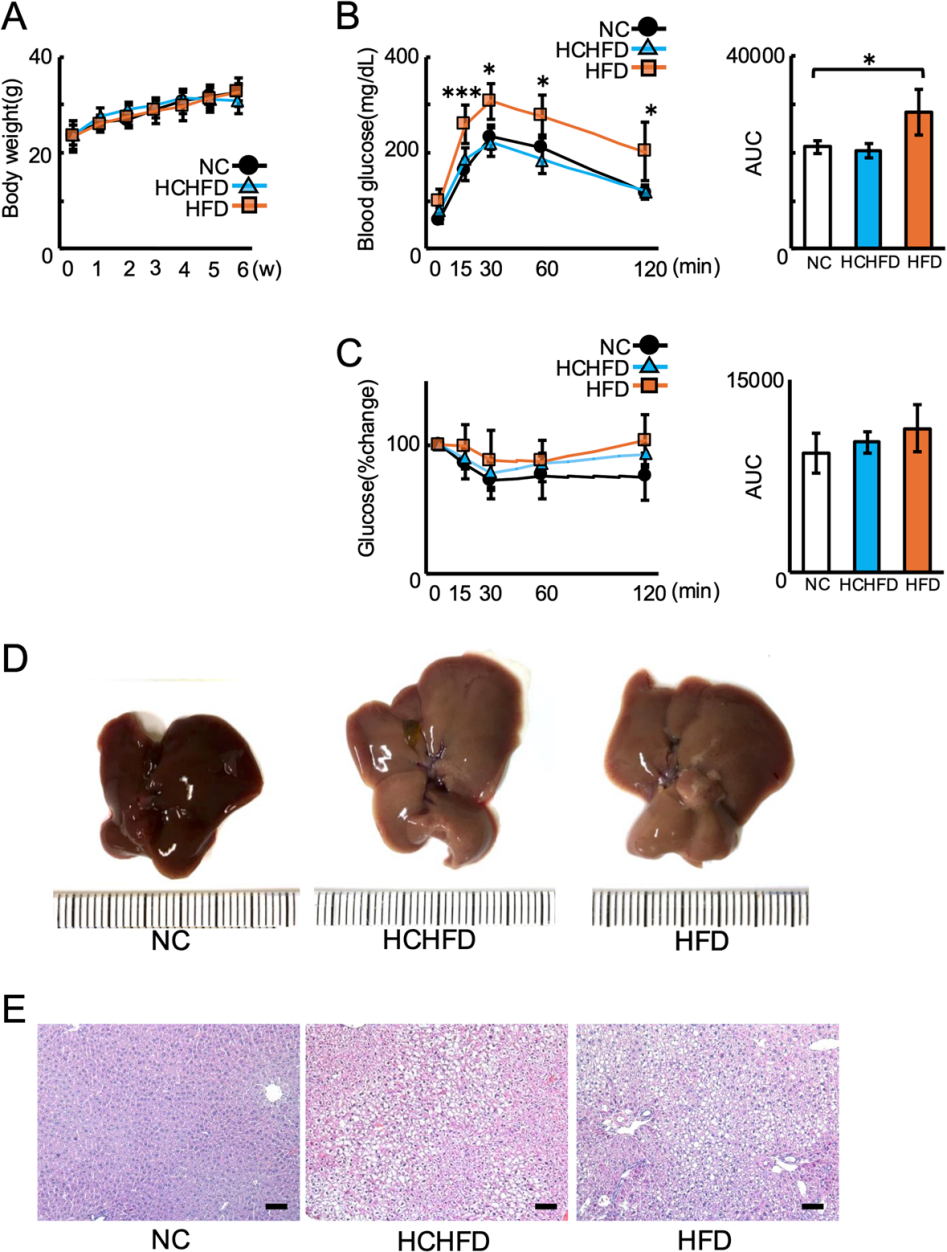
Body weight, glucose tolerance, insulin resistance, and liver steatosis in BALB/cAJcl mice fed each diet (*n* = 5). **A** Mouse body weights during the experiment (*n* = 5). **B** IPGTT (2 g/kg) was performed after overnight fasting for 6 weeks (*n* = 5). **C** ITT(1 U/kg) was performed after 6 h of fasting for 6 weeks (*n* = 5). **D** Representative images of the livers collected from NC-, HCHFD-, and HFD-fed mice at 6 weeks (*n* = 5). **E** HE staining of liver tissue from NC-, HCHFD-, and HFD-fed mice (scale bar = 100 μm) (*n* = 5). NC, normal chow diet; HCHFD, high-carbohydrate/high-fat diet; HFD, high-fat diet. *P < 0.05, **P < 0.005. All experiments were conducted independently at least three times with similar results

After a 6-week feeding period for each diet, we performed an intraperitoneal glucose test and an insulin tolerance test to assess whether the HFD and HCHFD caused glucose intolerance and insulin resistance. HFD mice did not show significant differences in body weight change or insulin resistance compared to NC mice (Fig. 1A and 1 C); however, they showed distinctive glucose intolerance (Fig. 1B). HCHFD mice did not show any differences in body weight, glucose intolerance, or insulin resistance (Fig. 1A-1C). Macroscopic findings showed that HFD induced obvious liver discoloration (Fig. 1D). Notably, HCHFD induced liver discoloration equivalent to that induced by HFD (Fig. 1D). In addition, histological analysis revealed marked lipid accumulation in both HFD and HCHFD mice (Fig. 1E). These findings suggest that HCHFD causes non-diabetic NAFLD, while HFD causes glucose intolerance accompanied by NAFLD in a short period in mouse studies.

### Ligature placement induced periodontal tissue destruction but did not affect glycometabolism in HCHFD-induced NAFLD mice

**Fig. 2:**
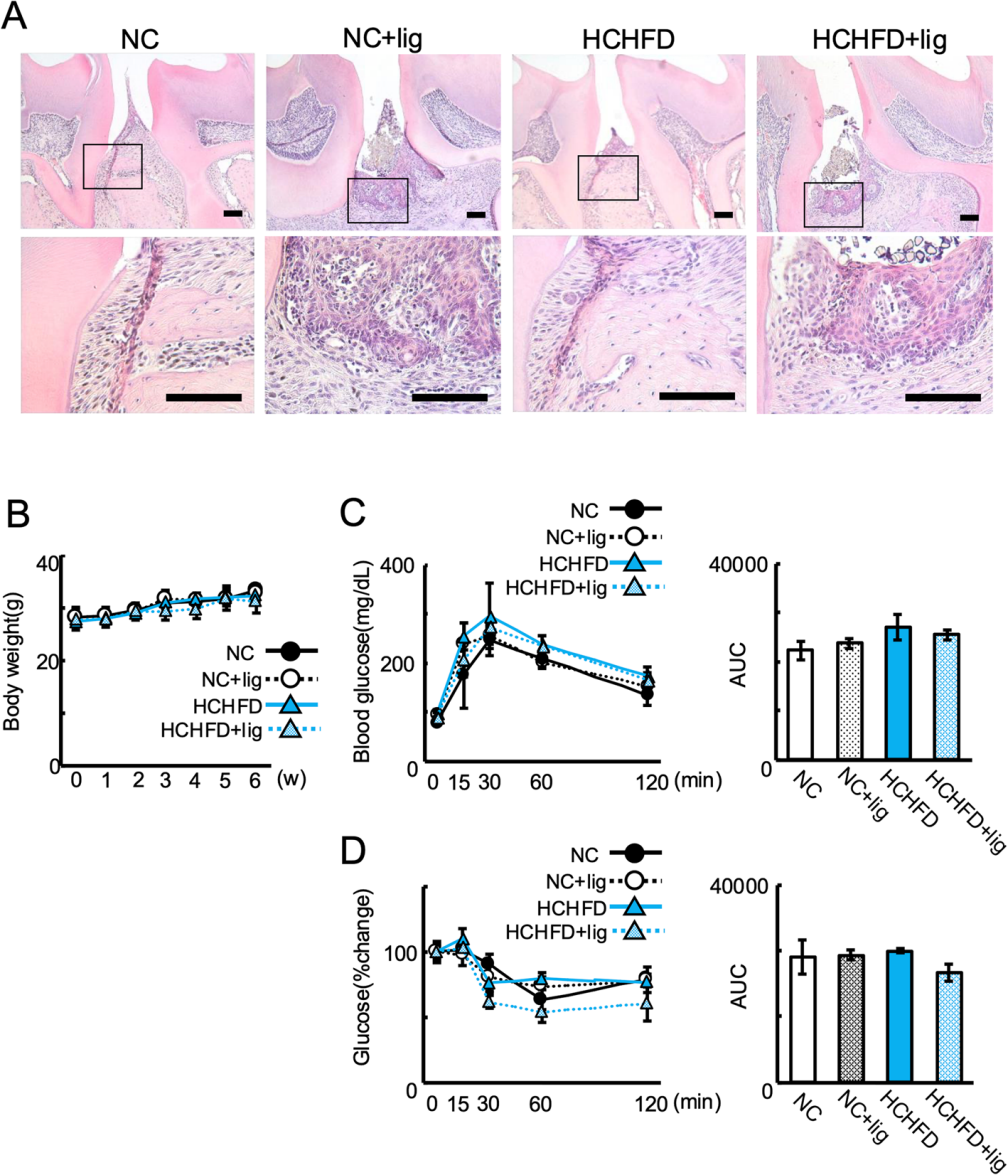
Effects of ligature-induced periodontitis in NC- and HCHFD-fed mice. **A** HE staining of periodontal tissues between first and second molars, 6 weeks after ligature placement. Upper panels show low- magnification images, with magnified views of the boxed regions shown in the lower panels (scale bar = 100 μm) (*n* = 3–4). **B** Body weight of NC, NC + lig, HCD, and HCD + lig mice during the entire experiment(*n* = 3–4). **C** IPGTT (2 g/kg) performed after overnight fasting at 6 weeks(*n* = 3–4). **D** ITT (1 U/kg) performed after 6 h fasting at 6 weeks(*n* = 3–4). NC, normal chow diet; NC + lig, normal chow diet with ligature placement; HCHFD, high carbohydrate/high-fat diet; HCHFD + lig, high carbohydrate/high-fat diet with ligature placement. All experiments were conducted independently at least three times with similar results

It has been reported that the placement of the silk ligature around the tooth leads to the accumulation of oral microorganisms, followed by inflammation and alveolar bone resorption in the ligature-induced periodontitis model [27].To determine whether periodontitis affects nondiabetic NAFLD, ligature-induced periodontitis was induced in a nondiabetic NAFLD mouse model induced by HCHFD (Fig. 1). In this ligature-induced periodontitis model, histological analysis showed that ligature placement induced alveolar bone resorption, epithelial hyperplasia, and inflammatory cell infiltration around the second maxillary molar in both NC and HCHFD mice (Fig. 2A). There was no significant difference in food intake among all the groups except the first day of the experiment (Supplementary Fig. 1). This indicated that the ligature placement did not impair food intake in the long run. To confirm that ligature-induced periodontitis did not affect glucose metabolism or lead to diabetes in the HCHFD group, IPGTT and ITT were conducted again in the four groups. Therefore, we confirmed that ligature-induced periodontitis did not affect glucose metabolism to cause diabetes in the HCD group. No significant differences in body weight, glucose intolerance, or insulin resistance were observed among the tested groups (NC, NC + lig, HCHFD, and HCHFD + lig) (Fig. 2B-2D). These data suggest that ligature placement induced periodontal tissue destruction but did not affect glucose intolerance or insulin resistance at 6 weeks.

### Ligature-induced periodontitis exacerbated lipid accumulation and caused fibrosis in HCHFD-induced NAFLD

**Fig. 3:**
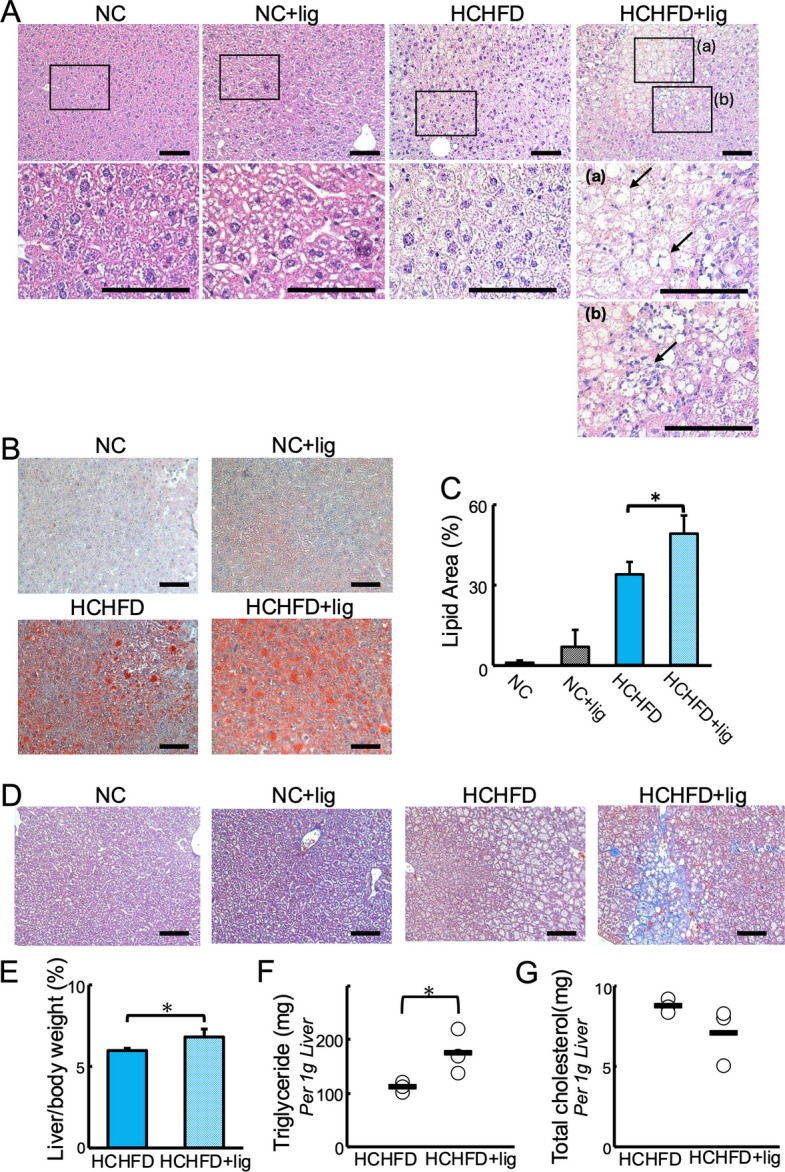
Effects of ligature-induced periodontitis on liver in NC, NC + lig, HCHFD, and HCHFD + lig mice. **A** HE staining of liver tissue from NC, NC + lig, HCHFD, and HCHFD + lig mice. Upper panels show low- magnification images, with magnified views of the boxed regions shown in the lower panels (scale bar = 100 μm) (*n* = 3–5). The arrows in (a) indicate ballooning cells. The arrow in (b) indicates inflammatory cells. **B** Oil red-O staining of liver tissue from NC, NC + lig, HCHFD, and HCHFD + lig mice (scale bar = 100 μm) (*n* = 3–5). **C** Lipid area quantified as the Oil Red O-stained area (%) using ImageJ software (*n* = 3–5). **D** Azan staining of liver tissue from NC, NC + ig, HCHFD, and HCHFD + lig mice (scale bar = 100 μm) (*n* = 3–5). **E** The liver weight to body weight ratio of HCHFD and HCHFD + lig mice (*n* = 3). **F** Weight of triglyceride (per 1 g of liver) (*n* = 3). **G** Weight of total cholesterol (per 1 g of liver) (*n* = 3). NC, normal chow diet; NC + lig, normal chow diet with ligature placement; HCHFD, high carbohydrate/high-fat diet; HCHFD + lig, high carbohydrate/high-fat diet with ligature placement. *P < 0.05. All experiments were conducted independently at least three times with similar results

Histological analysis showed marked lipid accumulation in HCHFD + lig mice compared to HCHFD mice (Fig. 3A). Oil red-O staining was performed to assess lipid accumulation in all the tested groups. The area of the lipid droplets was larger in the HCHFD + lig group than that in the HCHFD group (Fig. 3B,3C). Additionally, ballooning degeneration of hepatocytes and infiltration of inflammatory cells were observed in the HCHFD + lig group (Fig. 3A(a) and 3A(b)). Azan Mallory staining was performed to assess fibrosis in the liver. Fibrosis first arises around the central vein of the liver acinus and proceeds gradually to the portal vein and hepatic artery at the borders of the liver acini in NAFLD. It causes bridging fibrosis and eventually leads to liver cirrhosis in advanced stages. There were no signs of fibrosis in the NC, NC + lig, and HCHFD groups at week 6 (Fig. 3D). However, in the HCHFD + lig group, we observed distinctive fibrosis around the ballooning cells and inflammatory cells (Fig. 3D). Histological analysis showed that ligature-induced periodontitis exacerbated lipid accumulation and caused necroinflammatory changes and fibrosis in mice with HCHFD-induced fatty liver. Furthermore, quantitative evaluations were conducted among the HCHFD and HCHFD + lig groups to corroborate these results with the following experiments. The liver weight to body weight ratio was evaluated for each group, and shown to be higher in the HCHFD + lig group than the HCHFD group (Fig. 3E). Also, we measured the content of total cholesterol and triglycerides in the livers. There was no statistically significant difference in the content of total cholesterol between the two groups, but the content of triglyceride was higher in the HCHFD + lig group than the HCHFD group (Fig. 3F, 3G). Additionally, we evaluated the stage of fibrosis for each group [29]. Centrilobular fibrosis or interlobular fibrosis were seen in some cases of the HCHFD + lig group, while there were no signs of fibrosis in the HCHFD group (Table 4).

**Table 4:** Liver fibrosis stage

	**Stage0**	**Stage1**	**Stage2**	**Stage3**	**Stage4**
HCHFD	4	0	0	0	0
HCHFD ligature	1	2	1	0	0

## Discussion

In the present study, a non-diabetic NAFLD mouse model was established using an HCHFD diet. Ligature-induced periodontitis not only exacerbates fat accumulation but also causes interlobular fibrosis around the portal vein accompanied by hepatocellular injury in an established mouse model. However, there was no indication of glucose intolerance or insulin resistance due to ligature-induced periodontitis. Thus, these findings suggest that periodontitis may contribute to the onset of liver fibrosis in non-diabetic patients with NAFLD. This is notable considering that fibrosis is greatly involved in the prognosis of NAFLD [30–32]. Because the predominant drivers of the disease vary substantially among NAFLD patients [33], periodontitis might be critical in some cases. That being said, it should be noted that fibrosis caused by ligature-induced periodontitis was not to the point where it causes bridging fibrosis or elevates the level of serum AST or ALT, which are indexes of advanced liver fibrosis (Supplementary Fig. 2) [34]. Whether periodontitis affects the prognosis of NAFLD is still unknown. Further research to elucidate the potential role of periodontitis under non-diabetic conditions is needed, and we should cautiously assess its impact in clinical cases.

Several NAFLD mouse models have been established to reproduce the complex pathogenesis of clinical NAFLD with high accuracy [35]. These were also established to meet the needs of the research questions and purposes. Some models have been established to develop severe NAFLD in a short period of time (sacrificing the pathology that reflects human NAFLD) [36, 37] whereas others have focused on reproducing clinical features, including diabetes and obesity. The HCHFD-induced NAFLD model in the present study reflects the clinical features of the majority of NAFLD patients (i.e., non-diabetic NAFLD) and is therefore considered appropriate for assessing the impact of potential risk factors on this population. As mentioned previously, the complication rate of diabetes remains at 30%, even in advanced NAFLD with fibrosis. This model could also be used to elucidate unknown NAFLD-exacerbating pathways that are not involved insulin resistance. It has already been indicated that NAFLD, and consequently hepatocellular carcinoma, may develop through pathways independent of obesity or insulin resistance [22, 38]. Additionally, there may be other critical risk factors (besides periodontitis) that can be identified in this HCHFD-induced NAFLD model. Other critical risk factors for NAFLD besides periodontitis may be identified using this HCHFD-induced NAFLD model.

Although HCHFD successfully induced non-diabetic NAFLD in this study, there were some characteristics which are not always seen among diet-induced NAFLD mouse models in both HCHFD and HFD mice. For instance, there was no significant body weight gain in both HCHFD and HFD mice. We attribute this to the mouse strain utilized in the experiment. HFD certainly induced obesity accompanied by NAFLD in previous studies, but usually in cases where C57BL6 was utilized, which has been the most commonly used mouse strain. A previous study indicated that BALB/cAJcl used in this experiment is less likely to develop obesity compared to C57BL6 and other mouse strains [27]. Another study indicated that 10 week-feeing of HFD which contains almost the same percentage of fat as the present study did not induce obesity among BALB/cAJcl, instead developing steatosis and glucose intolerance. Thus, the results of the present study are compatible with the previous studies. Another feature is that it is a diet-induced NAFLD mouse model without insulin resistance or glucose intolerance induced by HCHFD. In this respect, the cholesterol included in HCHFD in this study could be what contributed to establish the non-diabetic NAFLD mouse model. There are some previous reports that support this idea. It is reported that NAFLD was correlated with cardiovascular disease events independently of the metabolic syndrome [39], and dietary cholesterol, though its influence has been downplayed recently [40, 41], is considered to be correlated with cardiovascular risk [41]. Moreover, Eng et al. reported that high-fat/high-fructose diet supplemented with 2% gm cholesterol (40% kcal fat) induced equivalent steatosis in mice while obesity, insulin resistance, and glucose intolerance were less pronounced compared with a similar diet without cholesterol [42]. While the amount of cholesterol used in the present study (0.15%gm) is less than that used in the previous study, it is possible that the cholesterol in HCHFD contributed to the non-diabetic NAFLD mouse model**.** Also, we measured the serum lipid levels of NC, HCHFD, and HFD mice. The serum levels of total cholesterol, free cholesterol, triglycerides, high-density lipoprotein cholesterol (HDL cholesterol) were higher in both HCHFD and HFD mice compared to NC mice (Supplementary Fig. 3 A, 3B, 3 C, 3E). However, the serum level of low-density lipoprotein cholesterol (LDL cholesterol) was higher only in HCHFD mice compared to NC mice (Supplementary Fig. 3D). LDL cholesterol is closely correlated with cardiovascular disease [43]. Dietary cholesterol could have compensated for comparable steatosis, which was induced by insulin resistance in HFD mice, through lipid metabolism changes. Moreover, we conducted the qPCR analyses of the mice livers of NC and HCHFD groups to measure inflammatory (IL-β, TNFα), fibrotic (TGFβ), lipogenetsis (Acc1, Glck) changes (Supplementary Fig. 4). Consequently, there were significant differences in mRNA expression of Acc1 and Glck between NC and HCHFD group (Supplementary Fig. 4 A, 4B). These results are consistent with liver steatosis in HCHFD mice livers. Nevertheless, underlying molecular mechanisms including hepatic insulin signaling which cause steatosis in HCHFD mice remains unknown in this study. It could be possible that HCHFD mice are similar to patients with hepatic insulin-sensitive NAFLD [44]

There are other mouse models which do not present glucose intolerance or insulin resistance in previous studies. For example, methionine- and choline-deficient diets result in excessive fat accumulation in the liver without insulin resistance [45]. However, this model deviates from the human NAFLD pathology in that it causes severe body weight loss and liver atrophy, which are not characteristics of clinical NASH [46]. In addition, methionine and choline deficiencies are not physiological. Another example is liver-specific PTEN KO mice, which show fat accumulation in the liver accompanied by fibrosis, a characteristic of clinical NASH without glucose intolerance [38]. However, whether PTEN-KO mice accurately reflect the clinical characteristics of non-diabetic NAFLD is questionable. The deletion of PTEN in the liver causes a decrease in serum insulin while simultaneously causing systemic hypersensitivity, thus compensating for the decline in serum insulin levels. Therefore, HCHFD-induced NAFLD mice simulate clinical non-diabetic NAFLD more closely than the existing NAFLD models. 

Nevertheless, HCHFD may not be appropriate for the assessment of anti-NAFLD drugs, because HCHFD alone does not cause fibrosis for at least 6 weeks. Since HCHFD feeding for more than 6 weeks was not conducted, it is unclear how long this non-diabetic NAFLD model lasts. Future studies on NAFLD will need to implement an extended experimental period to investigate its influence on ligature-induced periodontitis or other risk factors. To this end, the amount of carbohydrates in the current HCHFD may need to be reduced. We may also be able to evaluate the effect of periodontitis on advanced NAFLD with fibrosis under non-diabetic conditions by modifying HCHFD by adding more cholesterol. As we mentioned earlier, dietary cholesterol contributes to induce non-diabetic NAFLD. It is also known to cause liver damage including hepatocyte ballooning and fibrosis [39, 47, 48]. However, it also raises a concern that adding too much cholesterol is simply hepatotoxic and may not reproduce human diets linked to NAFLD [49]. A previous report suggested that adding 0.5% cholesterol could induce hepatic inflammation and fibrosis in mice [49]. While an amount of 0.2% cholesterol is still much higher than that generally ingested by humans, differences in cholesterol absorption between humans and mice may somewhat compensate for the gap, according to data in mice [49–52].

The results of this study indicate that ligature-induced periodontitis promotes steatosis and is involved in the onset of fibrosis in mice with HCHFD-induced non-diabetic NAFLD. However, the molecular mechanisms by which periodontitis affects nondiabetic NAFLD remain unclear. Previous studies have indicated that bacteria and their products are transported to the liver, where they cause NAFLD. Two possible pathways can be hypothesized based on previous studies. One of these is the oral–gut–liver axis. The oral-gut–liver axis is now widely accepted for periodontitis and liver diseases [53]. It has been reported that changes in the oral microbiota can alter the gut microbiota [54, 55]. Alterations in the gut microbiota could then cause disruption of the intestinal barrier (leaky gut syndrome) and thus the invasion of bacteria or endotoxins into the liver through the portal vein, which may eventually cause exacerbation of NAFLD [56, 57]. Recent studies have indicated that ligature-induced periodontitis alters the gut microbiota in mice [58, 59]. In addition, Hoyles et al. reported perturbations in the gut microbiota among women with non-diabetic NAFLD, highlighting the fact that gut microbiome-based therapies, including fecal microbiota transplantation, could represent an effective therapeutic approach [60]. Another possible pathway is the hematogenous metastasis of oral bacteria. Tsukasaki et al. detected oral bacteria in the liver of ligature-induced periodontitis group in mice [61]. Moreover, Miura et al. showed that there may be an NAFLD-exacerbating pathway via TLR4 in the liver that does not involve obesity or insulin resistance [22]. There is no disagreement that TLR4 plays a crucial role in NAFLD [62, 63]. TLR4 activation down regulates the transforming growth factor (TGF-beta) pseudoreceptor Bambi(BMP and activin membrane-bound inhibitor homolog) to sensitize hepatic stellate cells (HSCs) to TGF-beta-induced signals and enhance fibrosis [63]. TLR4 is closely associated with periodontal pathogens and gut microbiota. It recognizes lipopolysaccharide derived from gram-negative bacteria and pathogen associated molecular patterns [64–67]. Thus, periodontitis may cause the invasion of oral bacteria or endotoxins into the liver through intestinal barrier disruption or hematogenous metastasis, thus affecting NAFLD via TLR4 in the liver. Further research is required to understand the underlying mechanisms of periodontitis and non-diabetic NAFLD, and the HCHFD-induced model used in this study could contribute to this investigation.

## Conclusion

This study demonstrated that an HCHFD diet can be used to establish a non-diabetic NAFLD mouse model, successfully avoiding diabetic conditions such as glucose intolerance. Moreover, in this HCHFD-induced model, ligature-induced periodontitis exacerbated NAFLD, promoting steatosis and fibrosis under non-diabetic conditions.

## Supplementary Information

Supplementary Material 1.

Supplementary Material 2.

Supplementary Material 3.

Supplementary Material 4.

## Data Availability

All data generated or analyzed during this study are included in this published article.

## Abbreviations

NAFLD: Non-alcoholic fatty liver
HCHFD: High-carbohydrate/high-fat diet
HFD: High-fat diet
NAFL: Non-alcoholic fatty liver
NASH: Non-alcoholic steatohepatitis
MAFLD: Metabolic dysfunction associated liver disease
IPGTT: Intraperitoneal glucose test
ITT: Insulin tolerance test
HE: Hematoxylin eosin staining
NC: Normal chow
EDTA: Ethylenediaminetetraacetic
TLR4: Toll-like receptor4
TGF-beta: Ransforming growth factor-beta
Bambi: BMP and activin membrane-bound inhibitor homolog
HSC: Hepatic stellate cell
