# Supplementary figures and images for: Ligature-induced periodontitis exacerbates high-carbohydrate/high-fat diet-induced fatty liver in mice under non-diabetic conditions
Source: BMC Oral Health. 2026 Mar 4;26:944. doi: 10.1186/s12903-026-07992-6 (PMC13231554; doi:10.1186/s12903-026-07992-6)

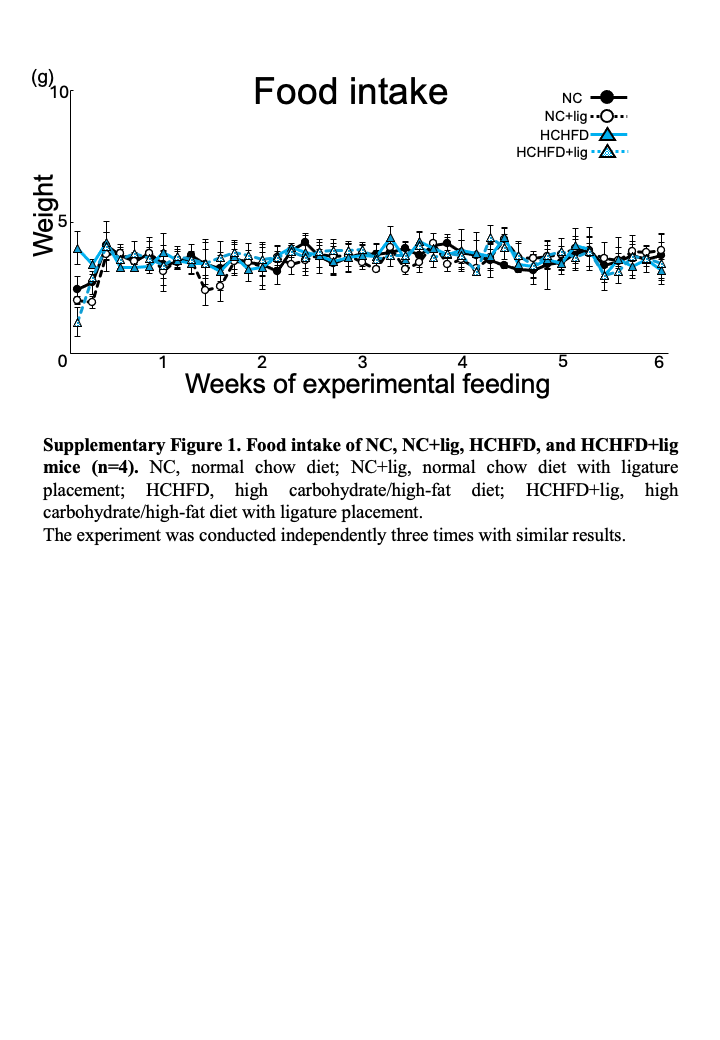

Supplement: Supplementary file 1 — Supplementary Material 1. [file 12903_2026_7992_MOESM1_ESM.tiff]

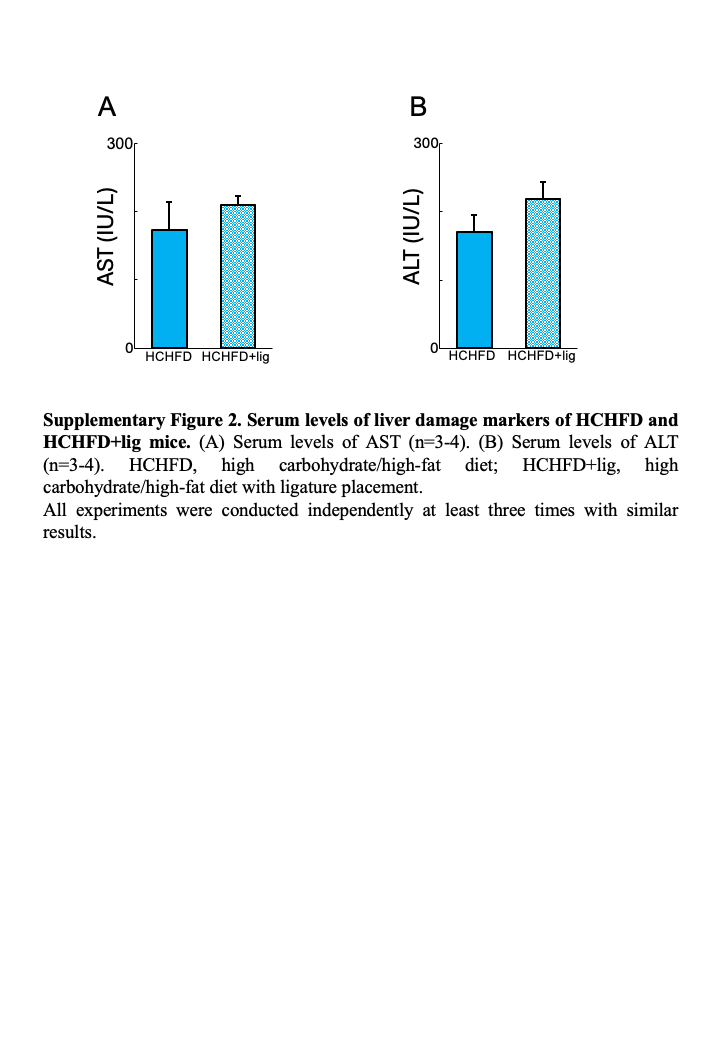

Supplement: Supplementary file 2 — Supplementary Material 2. [file 12903_2026_7992_MOESM2_ESM.tiff]

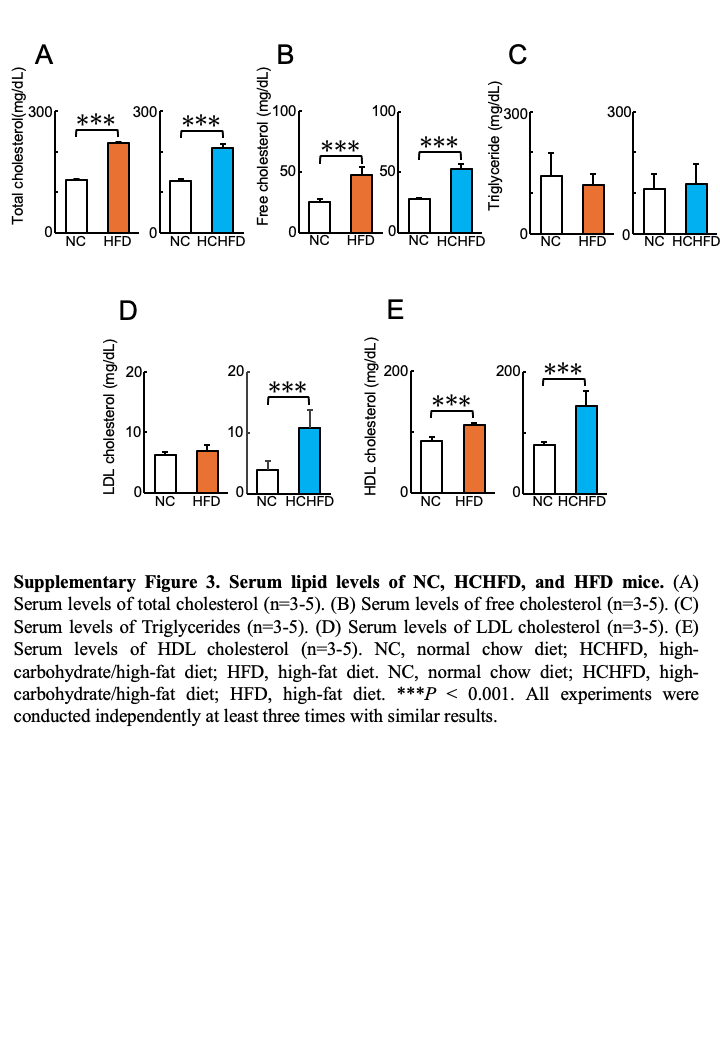

Supplement: Supplementary file 3 — Supplementary Material 3. [file 12903_2026_7992_MOESM3_ESM.tiff]

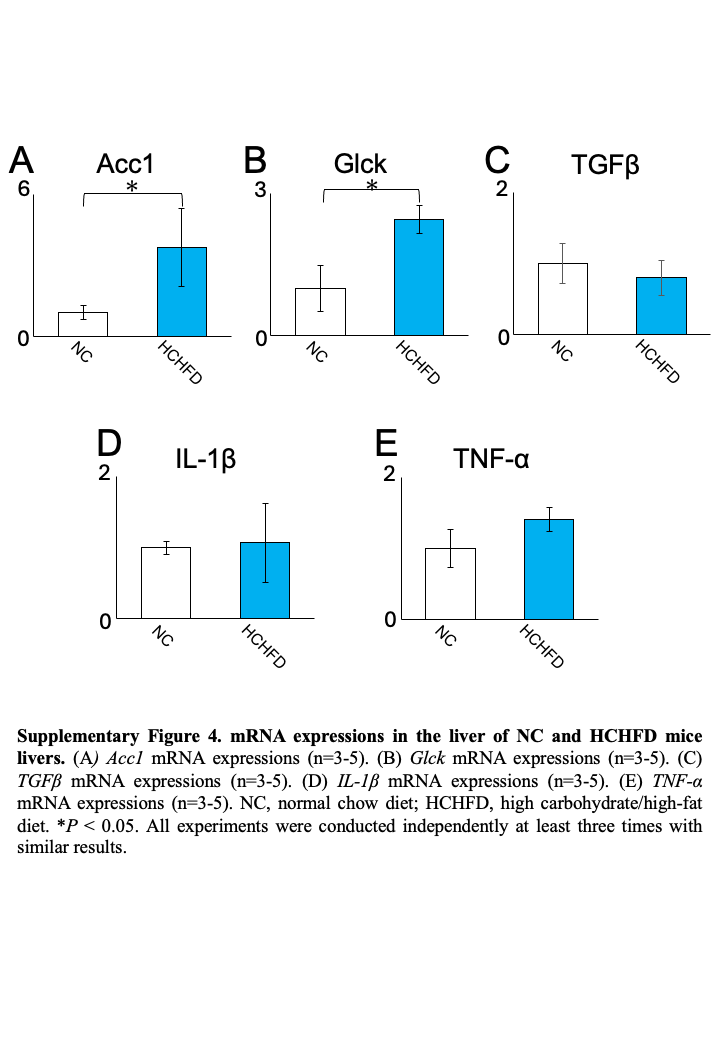

Supplement: Supplementary file 4 — Supplementary Material 4. [file 12903_2026_7992_MOESM4_ESM.tiff]
